# Improving CT-guided transthoracic biopsy diagnostic yield of lung masses using intraprocedural CT and prior PET/CT fusion imaging

**DOI:** 10.1186/s12890-022-02108-6

**Published:** 2022-08-13

**Authors:** Yue Lin, Yanyan Xu, Jie Lin, Liping Fu, Hongliang Sun, Zhenguo Huang, Bee Yen Ooi, Sheng Xie

**Affiliations:** 1 Department of Radiology, China-Japan Friendship Hospital, No. 2 Yinghua East Street, Chaoyang District, Beijing, 100029 China; 2 Department of Pathology, China-Japan Friendship Hospital, No. 2 Yinghua East Street, Chaoyang District, Beijing, 100029 China; 3 Department of Nuclear Medicine, China-Japan Friendship Hospital, No. 2 Yinghua East Street, Chaoyang District, Beijing, 100029 China; 4 Department of Radiology, Hospital Seberang Jaya, Jalan Tun Hussein Onn, Seberang Perai, 13700 Penang, Malaysia

**Keywords:** CT-guided transthoracic core-needle biopsy, Lung masses, FDG PET/CT, CT and PET/CT fused image -guided biopsy

## Abstract

**Objective:**

The purpose of this study was to evaluate the usefulness of intraprocedural CT and prior PET/CT fusion imaging in improving the diagnostic yield of CT-guided transthoracic core-needle biopsy (CNB) in lung masses.

**Methods:**

In total, 145 subjects with lung masses suspicious for malignancy underwent image-guided transthoracic CNB. According to imaging modality the subjects were divided into two groups. PET/CT images obtained no more than 14 days before the biopsy were integrated with intraprocedural CT images. The integrated or fused images were then used to plan the puncture sites. The clinical characteristics, diagnostic yield of CNB, diagnostic accuracy rate, procedure-related complications and procedure duration were recorded and compared between the two groups. Final clinical diagnosis was determined by surgical pathology or at least 6-months follow-up. The diagnostic accuracy of CNB was obtained by comparing with final clinical diagnosis.

**Results:**

145 subjects underwent CNB with adequate samples, including 76 in fusion imaging group and 69 in routine group. The overall diagnostic yield and diagnostic accuracy rate were 80.3% (53/66), 82.9% (63/76) for fusion imaging group, 70.7% (41/58), 75.4% (52/69) for routine group, respectively. In addition, the diagnostic yield for malignancy in fusion imaging group (98.1%, 52/53) was higher than that in routine group (81.3%, 39/48). No serious procedure-related complications occurred in both two groups.

**Conclusion:**

CNB with prior PET/CT fusion imaging is particularly helpful in improving diagnostic yield and accurate rate of biopsy in lung masses, especially in heterogeneous ones, thus providing greater potential benefit for patients.

## Introduction

Currently, CT-guided transthoracic core-needle biopsy (CNB) has emerged as a reliable and safe procedure for the diagnosis of indeterminate lung lesions, especially for the ones that are ineligible for surgical resection [1]. Excellent diagnostic accuracy and a relative low complication rate for this procedure have been confirmed in previous studies [2–4]. The diagnostic accuracy of CNB for malignant lung tumors varies between 92.7 and 99% [5–8]. However, false-negative rate may be substantially higher in large heterogeneous lesions or those with cystic and necrotic areas since only a small part of the lesion is obtained by CNB. In addition, adjacent atelectasis or obstructive pneumonia may also affect final biopsy results. For this reason, functional/metabolic methods, such as ^18^F-FDG PET/CT, which are helpful for identifying the hypermetabolic regions that represent actual biological behavior of the lesions for biopsy, are promising supplementary imaging tool for CNB [9–12].

The integration of intraprocedural CT and prior PET/CT images have been used in percutaneous biopsy of bone lesions, abdominal masses, mediastinal tumors and lung lesions [9–11, 13], PET/CT guided biopsy of suspected lung lesions was investigated in a previous study, but CNB under the guidance of PET/CT posed a certain radiation risk to the operator [14], moreover no consensus for its usefulness has been reached yet. However, to the best of our knowledge, there is no published information on prior PET/CT and CT fused images used in biopsy for lung masses. Also, we hypothesized that intraprocedural CT and prior PET/CT fused images are helpful in yielding a positive and accurate biopsy result. Thus, the purpose of this study was to establish the usefulness of intraprocedural CT and prior PET/CT fusion imaging in improving diagnostic yield of CNB in lung lesions by comparing to that of traditional CNB.

## Materials and methods

The institutional review board of the Ethical and Scientific Committees of China-Japan Friendship Hospital approved the present retrospective study and waived the requirement for informed consent for collecting data from the related patients. Written informed consent for CT-guided transthoracic biopsy had been obtained from all patients prior to performing the procedure.

### Study subjects

From January 2017 to December 2019, 145 patients with lung lesions that were suspicious for malignancy based on clinical and imaging findings were referred for CT-guided biopsy in our institution. The inclusion criteria were as follows: (1) lung mass or consolidation greater than 30 mm in short diameter; (2) 18 years or older; (3) definite clinical diagnosis by surgery or long-term follow-up (at least 6 months). In addition, the intra-procedural CT and prior PET/CT image fusions subgroup must have had their PET/CT examinations in our hospital as integration of two imaging modalities can not be performed in PACS otherwise, and the interval between PET/CT examination and CT-guided transthoracic CNB was no more than two weeks. The clinical characteristics of the patients were summarized in Table 1.

**Table 1:** Basic clinical characteristics of the patients undergoing CT-guided transthoracic core-needle biopsy

Variable		Fusion imaging group (N = 76)	Routine group (N = 69)	*P* value
Age(year)	Mean ± SD	65.1 ± 11.1	60.8 ± 12.6	0.031
Gender	Male/female	55/21	44/25	0.266
Smoking status	Ex- or current/never	10/24/42	26/11/32	0.002
Cancer history	Pulmonary/extra-pulmonary/no	0/9/67	0/3/66	0.102
Previous thoracic operation	Yes/no	2/74	4/65	0.339
Use of antiplatelet or anticoagulative drugs (e.g. aspirin, clopidogrel, warfarin, low molecular weight heparin, etc.)	Yes/no	14/62	17/52	0.362
Emphysema (on CT scan)	Yes/no	14/62	18/51	0.266
Location of consolidation or mass	Right/left/bilateral	42/29/5	33/33/3	0.472
Lobar involvement of consolidation or mass	< 1/2lobe/ > 1/2lobe/ > lobe	54/16/6	49/17/3	0.626
Lesion size (short diameter, cm)	Mean ± SD	5.18 ± 2.19	5.08 ± 1.88	0.770
Position for biopsy procedure	Supine/prone	48/28	44/25	0.939
Procedure duration (min)	Mean ± SD	8.7 ± 2.4	9.4 ± 3.4	0.198
The interval between PET/CT and CT (day)	Median ± IQR	5 ± 10	–	
Mean SUV_max_ value	Median ± IQR	8.0 ± 6.0	–	
^18^F-FDG uptake pattern	Uniform higher uptake/focal uptake/without uptake	29/46/1	–	
Operation/ follow-up*		23/53	16/53	0.337
The length of follow-up (month)	Median ± IQR	11 ± 7	10 ± 8	0.735
Complications	Pneumothorax/ intrapulmonary hemorrhage /hemoptysis	18/14/6	21/10/4	0.555
Note: SD, standard deviation; IQR, interquartile range; SUV, standardized uptake value				

### ^18^F-FDG PET/CT

^18^F-FDG PET-CT was performed using an integrated PET-CT scanner (GE Discovery ST; GE Healthcare Life Sciences, Chalfont, UK). All patients were fasted for at least 6 h before PET/CT scanning, and then 7.4 MBq/kg 18F-FDG was administered intravenously. (Atom Hi-Tech Co., Ltd., Beijing, China). One hour after 18F-FDG injection, the patient was supine and a PET scan was performed from the head to the mid-thigh. The attenuation correction and anatomical coregistration were derived from concomitant CT data without oral or intravenous contrast agents. The regional concentration of ^18^F-FDG was determined and expressed as the standardized uptake value (SUV). By using standard software tools provided with the PET/CT scanner, the SUV was adjusted for the injected dose of 18F-FDG and patient's weight. To minimize variation and ensure repeatability, the maximum SUV (SUVmax) was defined as the peak SUV of the pixel with the highest count in consecutive trans-axial scans. All PET/CT images will be automatically transferred to PACS after acquisition. During CNB procedure (detailed information described below), PET/CT images will be automatically matched with intraprocedural CT images using a built-in calibration software in PACS. The fusion images can also be adjusted manually by setting at least three points of common anatomical reference.

### Patient preparation and CT-guided transthoracic CNB procedure

Screening coagulation tests were ordered routinely before biopsy. Patients on antiplatelet or anticoagulant agents (e.g. aspirin, clopidogrel, warfarin etc.) were risk assessed independently by the interventional radiologists (with 24 and 13 years of experience respectively) and their referring physicians on the patients’ risk–benefit of medication cessation. The international normalized ratio needs to be maintained below 1.5, and the minimum platelet count maintained at 70,000/μL. Impaired coagulation status and platelet counts need to be corrected as much as possible if the above thresholds are not met [14]. In all cases, the interventional radiologists, thoracic surgeons and physicians jointly reviewed all available images including PET/CT and planned the biopsy approach.

Before CT-guided biopsy, patients were coached on slow breathing and held the breath at certain degree of inspiratory. All the planning and localization CT scans were carried out using the same 16-detector-row scanner (Aquilion 16; Canon Medical Systems). The following parameters were for the planning CT: scanning method = helical acquisition mode; tube currents = 50 mAs; tube voltage = 120 kVp; rotation time = 0.5 s; beam pitch = 1.0; imaging FOV = 400; slice thickness = 5 mm; the following parameters for the localization CT: scanning method = axial acquisition mode; tube currents = 50 mAs; tube voltage = 120 kVp; rotation time = 0.5 s; imaging FOV = 400; slice thickness = 4 mm. Scanning slices was limited to just cover the lesion in order to reduce radiation dose.

**Fig. 1:**
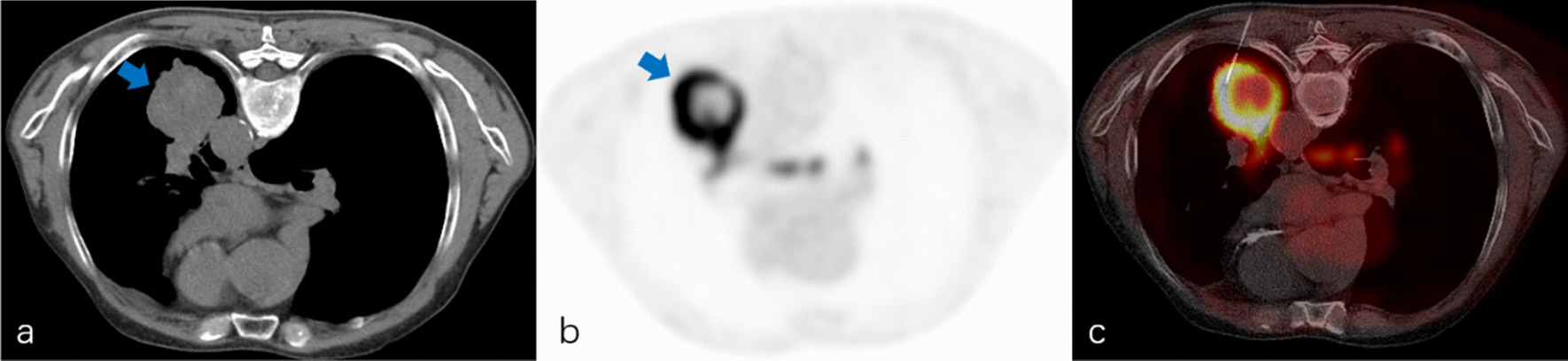
A 69-year-old man with suspected primary lung malignancy. **a** The intraprocedural non-contrast CT image (using mediastinal soft tissue window) showed a homogenous mass. **b** ^18^F-FDG PET/CT imaging showed a mass with uneven uptake (SUV_max_ = 12.8) (arrow) in the left lower lobe. **c** CT and prior PET/CT fused image showed FDG-avid peripheral region of the mass were targeted. Final surgery revealed lung large cell carcinoma with central necrosis

**Fig. 2:**
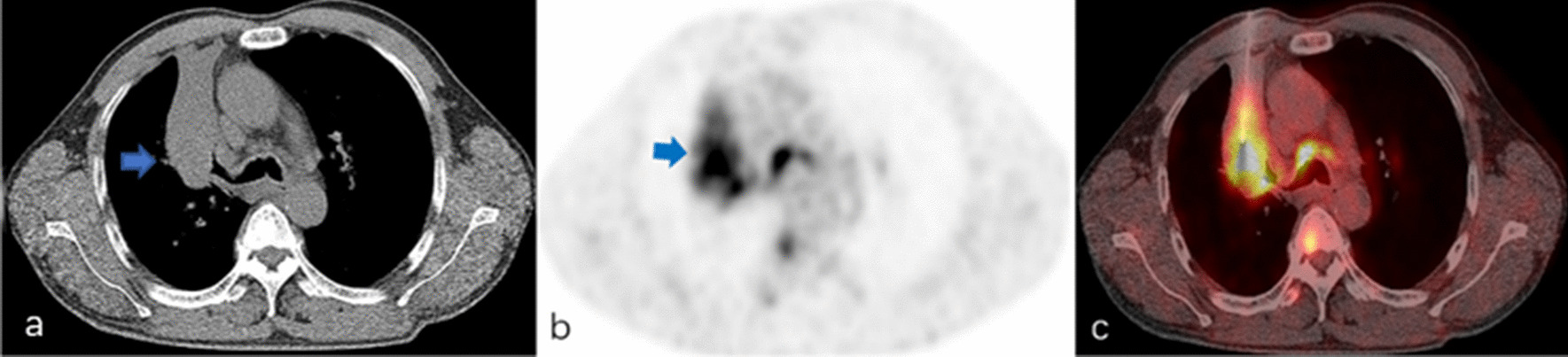
A 52-year-old man with suspected primary lung malignancy. **a** The intraprocedural non-contrast CT image (using mediastinal soft tissue window) showed a heterogenous mass in right upper lobe. **b** ^18^F-FDG PET/CT imaging showed heterogeneous uptake (SUVmax 10.2) in the mass with periphery hypometabolism. **c** CT and prior PET/CT fused image showed the biopsy needle punctured into hypermetabolic region of the mass. Surgical resection showed poorly differentiated lung adenocarcinoma with atelectasis

A planning CT scan (10-cm range) was performed before percutaneous needle insertion, and then the biopsy system including introducer needle (Cook Incorporated) and BioPince™ full core biopsy instrument (Argon medical devices, INC) was introduced in a stepwise manner under the guidance of images (CT images alone or CT and PET/CT fused images). An appropriate puncture point on a patient’s skin was marked to determine the shortest needle entry route while avoiding the inclusion of bullae and vascular structures. After local anesthesia with 2% lidocaine, a 10-cm-long 17-gauge percutaneous introducer needle was then advanced to puncture from the skin on the marked point without penetrating the parietal pleura. After confirming the direction of the tip of the puncture needle by the second CT scan (1.6 cm range), the needle was advanced further into the lesion (hypermetabolic area on fused images). Then the third CT scan was performed to confirm the final position of the tip of the needle before obtaining specimens coaxially. Two to three specimens were collected by BioPince™ full core biopsy instrument and subjected to histological evaluation for specific diagnosis (Figs. 1, 2). Finally, the fourth CT scan (the entire lung) was performed to rule out the presence of possible complications (e.g. pneumothorax, intrapulmonary hemorrhage, hemothorax, air embolism, etc.) after pulling out introducer needle. The CT-guided biopsy method was consisted with our previous studies. [15, 16] The severity of procedural complications was evaluated according to the Society of Interventional Radiology Standards of Practice Committee classification of complications [17], and procedure duration (defined as the interval time between the initial scout image and the end of the final CT scan) was also recorded. In addition, a repeat biopsy was considered when the patient’s clinical condition was obviously inconsistent with initial biopsy results (two patients) or when chemotherapy or targeted therapy regimens need to be adjusted (twenty patients). However, only the initial procedure result was recorded and analyzed in this study.

### Pathology results of biopsy

According to histopathological and microbiological analysis, the results of biopsy specimens were categorized as follows: malignancy; specific benign disease (e.g., hamartoma, infection, vasculitis); non-specific benign (fibrosis, necrosis or inflammation without identification of a specific disease) and nondiagnostic (only normal parenchymal cells and no infectious agents were found) [2], The final diagnosis was established through surgical resection or clinical and radiological follow-up (Once a month for first three month. If the lesion keeps stable, the interval will be adjusted to three months, if not, keep the interval before.) for at least 6 months following biopsy (13 subjects with malignancy died 6 months after biopsy). Diagnostic yield was defined as the percentage of specimens from biopsy with the diagnosis of malignancy and specific benign disease (N_biopsy_/N_final diagnosis_) [2]. In addition, all biopsy results mentioned above were classified as true or false diagnosis by whether biopsy specimen diagnoses correlated with the final diagnosis. Diagnostic accuracy rate was defined as the percentage of specimens with true diagnosis [3].

### Statistical analysis

According to CT-guided biopsy with or without PET/CT fusion imaging, all the subjects were divided into two groups: fusion imaging group and routine group. Continuous variables were expressed as mean ± standard deviation (SD) or median ± interquartile range (IQR). Independent-sample *t*-tests and Pearson’s chi-square tests were used for group comparisons of continuous and categorical variables. All statistical analysis was completed using SPSS 20.0. Statistical significance was set at *P* < 0.05.

## Results

Totally, 145 subjects (46 females, 99 males; mean age, 63.1 years; range, 22 ~ 84 years) who underwent CT-guided biopsy with adequate samples were enrolled, including 76 in fusion imaging group and 69 in routine group. Detailed clinical characteristics were summarized in Table 1. Of all subjects, 39 subjects had received surgery, 13 subjects died 6 months after biopsy for malignant disease progression, and 97 subjects had follow-up at least 6 months after initial biopsy.

The final diagnosis was obtained including 101 malignant lesions, 23 specific benign lesions, 21 non-specific benign lesions (Table 2). The overall diagnostic yield and diagnostic accuracy rate were 80.3% (53/66), 82.9% (63/76) for fusion imaging group, 70.7% (41/58), 75.4% (52/69) for routine group, respectively. In addition, the diagnostic yield for malignancy in fusion imaging group (98.1%, 52/53) was obviously higher than that in routine group (81.3%, 39/48).

**Table 2:** Association between diagnosis obtained from biopsy and final diagnosis

Lung lesion	Biopsy outcome		Final diagnosis	
	Fusion imaging group (N = 76)	Routine group (N = 69)	Fusion imaging group (N = 76)	Routine group (N = 69)
**Malignant**				
Lung cancer	50	37	51	46
Lymphoma	1	0	1	0
Metastatic carcinomas	0	1	0	1
Pulmonary sarcoma	1	1	1	1
**Benign**				
Tuberculosis	0	0	3	3
Organised pneumonia	0	0	4	0
Abscess	0	0	0	1
Aspergillosis	0	1	2	1
Infectious bronchiolitis	1	0	3	0
Interstitial pneumonia	0	0	1	3
Benign tumor	0	1	0	1
Mycobacterial infection	0	0	0	1
**Non-specific benign (fibrosis /inflammation /necrosis)**	15	19	10	11
**Non-diagnostic**	8	9	0	0

According to the Society of Interventional Radiology Standards of Practice Committee Classification [18], there were only minor complications, including localized pneumothorax (39/145; 26.9%), intrapulmonary hemorrhage (24/145; 16.6%) and hemoptysis (10/145; 6.9%), However, none of these patients needed further treatment. Furthermore, the rate of overall procedure-related complications in fusion imaging group was slightly lower than that in routine group (35.5% *vs* 44.9%). The procedure duration between two groups showed no significant difference (*P* = 0.198).

## Discussion

In this study, we found that 1) CNB was an effective and safe method in evaluating lung lesions with or without prior PET/CT fusion imaging, 2) intraprocedural CT and prior PET/CT fusion imaging can improve overall diagnostic yield and accuracy rate of CNB, especially in malignant lesions, 3) the overall complication rate in fusion imaging group was relatively lower, in contrast to routine group. The observations described above can be briefly summarized as follows: CNB with prior PET/CT fusion imaging is a feasible approach that improves diagnostic yield and accurate rate in lung masses (short diameter over 30 mm), thus providing greater potential benefit for patients.

The normal lung tissue is spongy and gas-filled, demonstrating periodic morphological changes during deflation and inflation, which is quite different from other solid organs (such as liver, bone). Large lung lesions (such as lung cancer) are prone to cause peripheral pneumonia, atelectasis, and even regional necrosis, which are hard to be distinguished from tumor on non-contrast CT images during biopsy. Furthermore, variation of breathing movement is another confounding factor. The interventional radiologist may have to repeatedly adjust the puncture site to avoid ribs or large vessels during biopsy. In addition, the pleural cavity and adjacent small airway are unavoidable structures during percutaneous lung biopsy, thus introducing a high risk of complications (such as pneumothorax, hemoptysis). In other words, percutaneous transthoracic biopsy is a relatively difficult procedure with high complication rate. Pneumothorax, intrapulmonary hemorrhage and hemoptysis were the most common complications in our sample, with an incidence of 26.9%, 16.6% and 6.9%, respectively. These results were similar to those of previous studies [6, 7].

As we all know, ^18^F-FDG PET/CT provides metabolic information related to disease and has shown a great advantage in targeting potential abnormal disease, especially malignant tumors [2–4, 9–12]. In the current study, only the subjects with lung mass or consolidation greater than 30 mm in short diameter were enrolled. Generally, the larger the lung lesion, the more likely it is to be heterogeneous (necrosis, fibrosis, atelectasis, etc.), resulting in an uneven FDG uptake [19]. Theoretically, intraprocedural CT and prior PET/CT fusion imaging can identify the lesion site and its extent better, thus reducing a preselection bias for biopsy-site and improving diagnostic performance. However, FDG avidity could also be observed in some benign tumors, infective and inflammatory conditions that are difficult to distinguish from each other [20]. It is no wonder that even though FDG-avid regions were targeted, some false negative biopsy results were obtained in the current study. Actually, similar conditions were also reported in previous studies [9–12]. This might be one of reasons that no consensus has been reached yet for its usefulness. A recent study reported that CNB under the guidance of PET/CT was superior to CT-guided biopsy, PET/CT guided biopsy of lung lesions led to fewer inconclusive biopsies in comparison with CT guided biopsy, with similar complication rates. However, PET/CT guided biopsy had a certain radiation risk to the operator. In addition, the size of the lesion in that study was not limited to mass or consolidation. In clinical work, the risk of necrosis and bleeding of smaller nodules was low. Generally, significant pathological results can be obtained according to CT guidance biopsy. PET/CT guided biopsy was not necessary in these cases. Their research results also showed that lesions that required rebiopsy in the PET/CT group had a greater average size than those on the CT group (9.6 × 4.6 cm, p = 0.04). In our study, we enrolled masses or consolidation larger than 3 cm. In these cases, PET/CT images were necessary to guide biopsy [17].

Interestingly, even though CNB of lung lesions is a high-risk procedure, but no severe complications developed. Furthermore, intraprocedural CT and prior PET/CT fused imaging allows for more accurate identification for FDG-avid lesions that more accessible to biopsy, reducing the likelihood of complications and procedure duration. So, the procedure duration of fusion imaging group was slightly shorter than that of routine group (8.7 min vs. 9.4 min), although no significant difference was observed.

There are several limitations in the current study. First, this is a retrospective study performed in a single center. Second, currently, PET/CT examination is not a routine item covered by health insurance, thus introducing a potential selection bias for the patients in fusion image group, in other words, fusion imaging group has significantly fewer benign lesions than routine group. Third, even though the follow-up duration was relatively limited compared to previous studies [9–12], 97(66.9%) subjects were followed up for more than 10 months, the follow-up duration of previous studies, and hence a definite clinical diagnosis could be obtained to some extent.

## Conclusion

CNB with prior PET/CT fusion imaging is particularly helpful in improving diagnostic yield and accurate rate of biopsy in lung masses, especially in heterogeneous ones, thus providing greater potential benefit for patients.

## Data Availability

The imaging datasets used and/or analyzed during the current study are not publicly available due to the policies of the institutions. The research datasets used and/or analyzed during the current study are available from the corresponding author upon reasonable request.

## Abbreviations

CNB: CT-guided transthoracic core-needle biopsy
PACS: Picture archiving and communication system
SUV: Standardized uptake value
FOV: Field of view
SD: Standard deviation
IQR: Interquartile range
